# Phononic-Crystal-Based Particle Sieving in Continuous Flow: Numerical Simulations

**DOI:** 10.3390/mi13122181

**Published:** 2022-12-09

**Authors:** Laixin Huang, Juan Zhou, Deqing Kong, Fei Li

**Affiliations:** 1 Paul C. Lauterbur Research Center for Biomedical Imaging, Shenzhen Institutes of Advanced Technology, Chinese Academy of Sciences, Shenzhen 518055, China; 2 Muroran Institute of Technology, Muroran 050-8585, Hokkaido, Japan

**Keywords:** phononic crystal, particle sorting, acoustic radiation force, acoustic streaming, microfluidics

## Abstract

Sieving specific particles from mixed samples is of great value in fields such as biochemistry and additive manufacturing. In this study, a particle sieving method for microfluidics was proposed based on a phononic crystal plate (PCP), the mechanism of which originates from the competition between the trapping effect of the resonant PCP-induced acoustic radiation force (ARF), disturbance effect of acoustic streaming (AS), and flushing effect of the continuous inlet flow on particles suspended in microfluidic channels. Specifically, particles with different sizes could be separated under inlet flow conditions owing to ARF and AS drag forces as functions of the particle diameter, incident acoustic pressure, and driving frequency. Furthermore, a comprehensive numerical analysis was performed to investigate the impacts of ARF, AS, and inlet flow conditions on the particle motion and sieving efficiency, and to explore proper operating parameters, including the acoustic pressure and inlet flow velocity. It was found that, for each inlet flow velocity, there was an optimal acoustic pressure allowing us to achieve the maximum sieving efficiency, but the sieving efficiency at a low flow velocity was not as good as that at a high flow velocity. Although a PCP with a high resonant frequency could weaken the AS, thereby suiting the sieving of small particles (<5 μm), a low channel height corresponding to a high frequency limits the throughput. Therefore, it is necessary to design a PCP with a suitable resonant frequency based on the size of the particles to be sieved. This investigation can provide guidance for the design of massive acoustic sorting mi-crofluidic devices based on phononic crystals or acoustic metamaterials under continuous flow.

## 1. Introduction

Sieving specific particles or cells from mixed samples is important in many fields, including biomedical research, chemical analysis [1], and wastewater treatment [2]. For example, the diagnosis and treatment of HIV rely on the isolation of CD4+ T lymphocytes from human blood [3]. Likewise, the diagnosis and treatment of malaria rely on the separation of parasite-infected red blood cells from uninfected cells [4]. Conventional methods, such as centrifugation [5], filtration [6], and solvent addition [7] have the advantages of fast, high-volume processing. However, these methods are often insufficient to fully satisfy increasingly stringent requirements such as small differences in the density and size of mixed particles, biological compatibility, continuous processing, and device miniaturization [8].

Recently, a sorting method combining microfluidics and various physical fields such as light, electricity, magnetism, and sound has been developed and has provided efficient solutions for multiple separation scenarios [9,10,11,12,13,14]. For example, cells have been sorted from flowing samples using the trapping force of a highly focused laser beam. It was found that there is an optimal balance between the optical trapping force and sample flow rate that may be used to achieve high separation efficiency [15]. In addition, conducting polydimethylsiloxane (PDMS) composite electrodes were embedded into the sidewall of a PDMS channel, and polystyrene particles with diameters of 5, 10, and 15 μm were separated using the dielectrophoretic force generated by the electric field created by the electrodes [16]. Additionally, microfluidic systems with high-intensity and high-gradient magnetic fields were used to sieve nanoparticles of different sizes [17].

In contrast to the above methods, acoustic waves can be used to sieve particles with different densities and compressibilities, in addition to relying on size differences. With the advantages of being label-free, noncontact, and biocompatible, the acoustic sorting of particles relies on the acoustic radiation force (ARF) and acoustic streaming (AS) to control the movement of the particles [18,19,20]. For example, an acoustic surface wave device was used to generate a standing wave and separate exosomes or circulating tumor cells from blood [21,22]. In addition, a bulk standing wave was used to sort polystyrene and PDMS particles of the same size but with different densities and sound speeds. Because the acoustic contrast factors of these two types of particles were different, they were arranged at the pressure- and anti-nodes of the standing wave field, respectively [23]. An array of acoustic microstreaming traps was also used for the parallel separation of tumor cells from biological samples [24].

Typically, existing acoustic separation methods such as surface wave devices rely on a slender channel with a half-wavelength width and require sheath flow to focus the sample, thus limiting the throughput. Phononic crystals are artificial structures with a periodic distribution of acoustic parameters such as mass density and sound speed, which can control the transmission of acoustic waves. Modulating the acoustic field based on phononic crystals may provide a flexible tool for acoustic sorting. For example, the acoustic radiation force and acoustic streaming field in a droplet allow the separation of suspended microparticles based on the scattering of surface acoustic waves by asymmetric pillar-type phononic crystals [25]. By designing a two-dimensional linear defect waveguide, solid particles with different sizes and densities were separated in air in a noncontact manner [26]. Previously, we constructed a scalable and tunable “acoustic sieve” based on the balance of the gravity and vertical gradient radiation force of a resonant phononic crystal plate (PCP), which enabled the sieving of massive subwavelength particles with a diameter range of 100 to 400 μm [27]. Because acoustic sieving was realized under the no-flow condition wherein the sample was initially stationary, this method lacked the capability of continuous processing, compared to sorting methods under flow conditions. In addition, in the previous study concerning an “acoustic sieve” with a working frequency of 1.28 MHz, the ARF and gravity dominated the dynamics of the manipulated particles larger than 100 μm, while the effect of the AS drag force on the particle motion was ignored. As the particle size was decreased to less than 10 μm, the gravity effect could be ignored, while the contribution of AS to the particle’s acoustophoretic motion had to be considered. Therefore, exploiting the dynamics of particles smaller than 10 μm driven by the PCP-induced ARF and AS under the inlet flow conditions could provide a low-cost, disposable, and batch-processing tool for massive acoustic sieving processes. Because many parameters are involved in acoustic sieving, a comprehensive numerical simulation is performed in this study to examine the feasibility of this method for particle sieving and to explore suitable parameters to provide theoretical guidance for the design of microfluidic devices and experiments.

## 2. Materials and Methods

**Figure 1:**
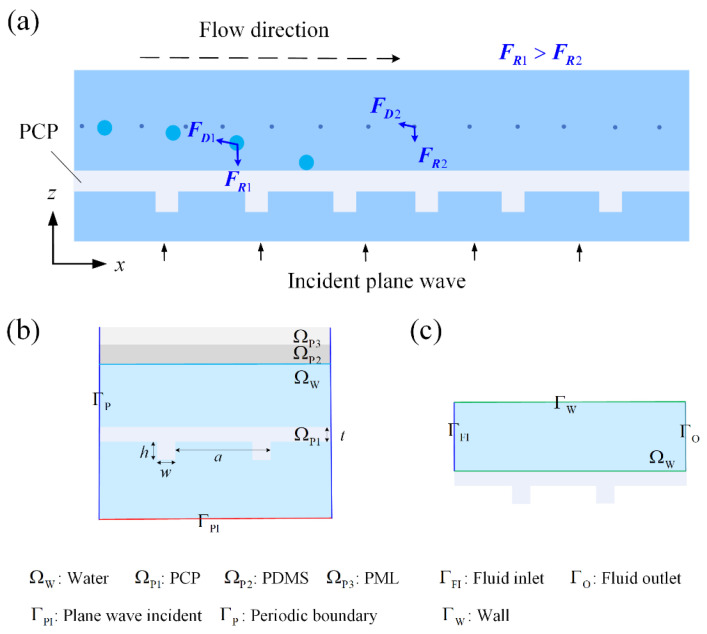
(**a**) Schematic of PCP-based particle sieving. The acoustic radiation force produced by a resonant PCP traps large particles from mixed samples under the flow condition. Simulation model and boundary conditions for calculating (**b**) the first-order acoustic field and (**c**) streaming field.

The working principle of acoustic sieving is illustrated in Figure 1a. A PCP is a thin plate with a periodic grating on one side of the surface. Acoustic transmission enhancement (ATE) is induced under a normal incident plane wave at the resonant frequency of the PCP. The match between the dispersion curves and the blue shift of the transmission peak, observed for different angles of the incident wave, further confirmed that the ATE was correlated to the excitation of non-leaky $A_{0}$ Lamb waves in the PCP [28,29,30,31]. Thereafter, an acoustic field with exponential decay and high gradient distribution along the z-direction is generated around the PCP. Because of the reflection or absorption of sound energy by the particles, the acoustic field further exerts an ARF $F_{R}$ on the particles in it [32]. $F_{R}$ can trap suspended particles on the PCP surface. When the acoustic characteristics of the particle, such as the particle density and sound speed, remain unchanged, $F_{R}$ is proportional to the cube of the particle radius [33]. Therefore, when mixed particles of different sizes but similar acoustic characteristics flow over the PCP with an inlet flow velocity of $u_{0}$, the acoustic trapping of larger particles can be realized by adjusting the incident acoustic pressure $p_{0}$, while the smaller particles flow out of the channel with the inlet flow to realize the separation of different particles, as shown in Figure 1a.

The particles are mainly subjected to three types of forces in the channel: ARF, AS drag force, and inlet flow drag force. In addition to the ARF, the viscous dissipation on the PCP surface, under a resonant acoustic field, induces enhanced near-boundary AS [34,35], which generates a drag force on the microparticles to affect their motion. Because particle sieving is performed under inlet flow conditions, the particles are also subjected to the inlet flow drag force. In this study, the three force-driven motions of particles are numerically simulated to determine suitable parameters for particle sieving under different inlet flow velocities $u_{0}$ and incident acoustic pressures $p_{0}$.

The simulation was implemented using COMSOL Multiphysics 5.3a (COMSOL, Stockholm, Sweden). Coupled “Thermoviscous Acoustics, Frequency Domain” and “Solid Mechanics” modules were used to compute the acoustic field in water and resonant displacement of PCP. The simulation model and boundary conditions are shown in Figure 1b. Particles away from the PCP were difficult to trap because of the localized characteristics of the acoustic field in the z-direction. Therefore, the channel height was set to 100 μm. In addition, a layer of PDMS with thicknesses of 20 μm and a perfect match layer with a thickness of 10 μm were applied at the top of the channel to simulate the acoustic reflection and absorption in the PDMS. The governing equations of the first-order acoustic field can be expressed as [36]:

(1) $$\rho_{0}\frac{\partial u_{1}}{\partial t}=-\nabla p_{1}+\mu \nabla^{2}u_{1}+\left(\eta +\frac{1}{3}\mu \right)\nabla \left(\nabla \cdot u_{1}\right)$$

(2) $$\frac{\partial \rho_{1}}{\partial t}=-\rho_{0}\nabla \cdot u_{1}$$

(3) $$\rho_{0}C_{p}\frac{dT}{dt}-\alpha_{0}T\frac{dp_{1}}{dt}=\nabla \cdot \left(k_{th}\nabla T\right)$$

where $\rho_{0}$ and $\rho_{1}$ represent the density of water and its first-order perturbation, respectively; η and μ are the bulk and shear viscosities of water, respectively; $k_{th}$ is the thermal conductivity, $C_{p}\;$ is the specific heat capacity, $\alpha_{0}$ is the thermal expansion coefficient and T is the temperature. $p_{1}$ and $u_{1}$ are the first-order acoustic pressure and acoustic particle velocity, respectively.

In this study, the resonant frequency f of the PCP was 4 MHz. The wavelength in water was $\lambda =c_{0}/f=375\;\mu m$, where $c_{0}=1500\;m/s$ denotes the speed of sound in water. Because the diameter of the particles in this study is much smaller than λ, we used Gor’kov ‘s theory [33] to calculate the $F_{R}$ exerted on a particle with radius r:

(4) $$F_{R}=-\nabla U$$

(5) $$U=2\pi r^{3}\rho_{0}\left(f_{1}\frac{\langle p_{1}^{2}\rangle }{3\rho_{0}^{2}c_{0}^{2}}-f_{2}\frac{{\langle \left|u_{1}\right|\rangle }^{2}}{2}\right)$$

(6) $$f_{1}=1-\frac{\kappa_{p}^{2}}{\kappa_{0}^{2}},\;f_{2}=2\frac{\rho_{p}-\rho_{0}}{2\rho_{p}+\rho_{0}}$$

where ω=2πf denotes the angular frequency of the acoustic wave, 〈·〉 represents the time-average operator. $\kappa_{p}$ and $\rho_{p}$ represent the compressibility and density of the particles, respectively.

Because of the microscale characteristic sizes, including the particle diameter and channel cross-section, the flow in this study exhibits a low Reynolds number (Re << 1). In addition, the compressibility of water can be neglected for acoustic microstreaming [37,38] because the streaming velocity, with a typical magnitude of tens of micrometers per second, is much smaller than the sound speed in water (1500 m/s). Therefore, we used the “Creep Flow” module to compute the flow field, which is equivalent to the well-known second-order perturbation theory for AS under the condition of incompressible flow, expressed as $\nabla \cdot \langle \rho_{1}u_{1}\rangle =0\;$ [39]. The total flow field is the superposition of the inlet flow and AS. The computation model is shown in Figure 1c. The governing equations for the creep flow, ignoring the inertia term, can be expressed as [34,37,40]:

(7) $$\nabla p_{2}=\mu \nabla^{2}u_{f}+F$$

(8) $$\rho_{0}\nabla \cdot u_{f}=0$$

(9) $$F=-\langle \rho_{0}u_{1}\nabla \cdot u_{1}+u_{1}\cdot \nabla u_{1}\rangle$$

where $u_{f}$ and $p_{2}$ are the velocity and pressure of the second-order flow field, respectively, and F is the volume force. Both the inlet and outlet were set to fully developed velocity boundary conditions, the velocity magnitude was set to $u_{0}$, and the top and bottom walls were set as no-slip boundaries. 

The drag force $F_{D}$ exerted on the spherical particles in the flow field can be expressed as:

(10) $$F_{D}=6\pi \mu r\left(u_{f}-u_{p}\right)$$

where $u_{p}$ is the velocity of the particle. The motion of the particles is described by Newton’s second law:

(11) $$F_{R}+F_{D}=m_{p}\frac{du_{p}}{dt}$$

where $m_{p}$ is the mass of the particles. Particle motions were simulated using the “Particle Tracing for Fluid Flow” module. 

## 3. Results and Discussion

### 3.1. Transmission Spectrum of the PCP and Acoustic Field

First, the transmission spectrum was plotted to determine the resonant frequency of the PCP. When the traveling plane wave encounters the PCP, reflected and transmitted waves are generated. The incident wave intensity is calculated as $I_{in}=\int {\left|p_{0}\right|}^{2}/2\rho_{0}c_{0}\cdot dl$ where the integration is performed along the boundary $\Gamma_{\mathrm{PI}}$. The transmitted wave intensity is calculated as $I_{trans}=\int {\left|p\right|}^{2}/2\rho_{0}c_{0}\cdot dl$ where the integration is performed along the boundary, namely, the convergence of zones $\Omega_{W}$ and $\Omega_{P2}$, and p is the acoustic pressure at the boundary. The transmission T is defined as the ratio of the transmitted wave intensity to the incident wave intensity, that is, $T=I_{trans}/I_{in}$. When a traveling plane wave is an incident at the resonant frequency of the PCP, $I_{trans}$ and T reach the maximum values, and a peak occurs in the transmission spectrum. In this study, the thickness of the PCP was t=50 μm, the grating width and thickness were w=h=50 μm, and the lattice constant was a=300 μm. The material was stainless steel, the density was $\rho_{s}=7760\;\mathrm{kg}/m^{3}$, and the longitudinal and transverse wave velocities were $c_{l}=6010\;m/s$ and $c_{t}=3320\;m/s$, respectively.

**Figure 2:**
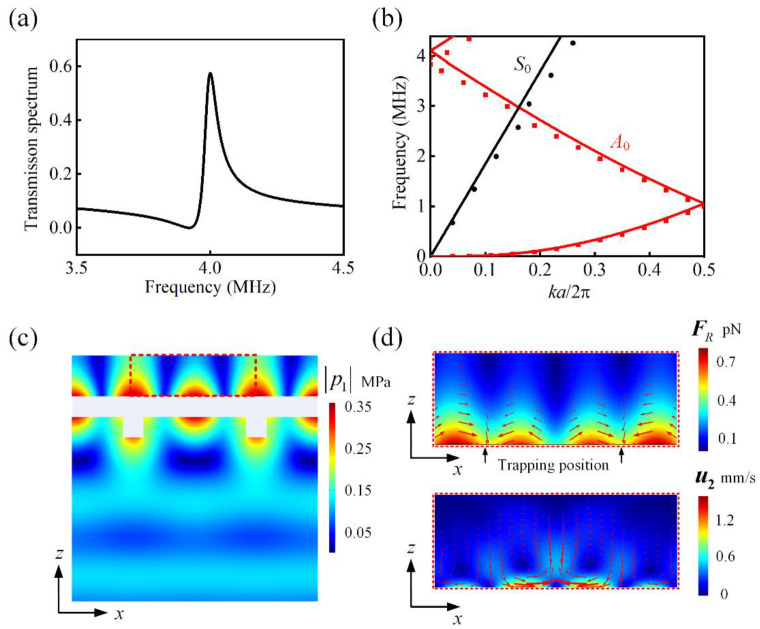
(**a**) Transmission spectrum of the PCP with t=50 μm, w=h=50 μm, and a=300 μm. (**b**) Numerical dispersion curves (black and red dots) for the PCP sample with a one-period structure. For comparison, the simply folded dispersion curves for the uniform plate are plotted as lines with the same color. Simulated amplitudes of the (**c**) first-order acoustic pressure and (**d**) acoustic radiation force $F_{R}$ and velocity of AS $u_{2}$.

The transmission spectrum is shown in Figure 2a. Evidently, transmission enhancement occurs at 4 MHz, which is the resonant frequency of the PCP. The numerical dispersion curve of the $A_{0}$ mode Lamb wave for one period of the structured plate further validates the resonant mode of the PCP. As illustrated in Figure 2b, the frequency at normal incidence (k=0) was 4 MHz, corresponding to the transmission peaks in Figure 2a. For the dispersion curve of the $S_{0}$ mode Lamb wave, the frequency is equal to zero at k=0, which indicates that the 4 MHz $S_{0}$ mode Lamb wave cannot be excited under normal incidence. In addition, the PCP resonant frequency is much lower than the cutoff frequency of the non-zero-order Lamb waves (symmetric modes $S_{n}$ and antisymmetric modes $A_{n}$, n=1,2,3,…) [28]. Therefore, only the $A_{0}$ mode Lamb wave was excited under the normal incident plane wave at the PCP resonance frequency to generate the transmission peak.

Next, the first-order acoustic pressure field, ARF, and AS distributions around the PCP at 4 MHz were computed. The pressure field under the incident acoustic pressure $p_{0}=100\;\mathrm{kPa}$ is shown in Figure 2c. The maximum acoustic pressure was 360 kPa. In the x-direction, the acoustic field exhibits characteristics of periodic distribution, similar to the standing wave field. However, in the z-direction, the acoustic pressure decays rapidly and exhibits a strong gradient. The distribution of the ARF exerted on the particles in the acoustic field is shown in Figure 2d. The parameters of the particles were derived from Escherichia coli, the equivalent radius [41] was $r_{p1}=1.15\;\mu m$, the density was $\rho_{p}=1160\;\mathrm{kg}/m^{3}$, and the sound velocity was $c_{p}=1600\;m/s$. It can be observed that the ARF is highly localized on the surface. Although the force in the z-direction is not always a trapping force, the ARF pushes the particles toward the trapping position owing to the force of the x-direction component, as shown by the black arrow in Figure 2d. There are two locations between any two neighboring gratings where particles are trapped on the PCP surface. From the AS distribution in Figure 2d, it can be observed that the AS on the surface of the PCP presents a vortex-like flow, which tends to entrain smaller particles into the center of the vortex, thus affecting the separation of the particles.

It is noted that the AS in Figure 2d shows a high flow velocity on the order of mm/s compared with a typical AS velocity with a magnitude of tens of μm/s in microfluidics, such as that of the Rayleigh–Schlichting streaming (RSS) driven by a traditional bulk standing wave [42]. The main reason for this is the existence of both tangential and normal vibrations on the surface of the PCP, which provide a larger volume force and induce a stronger AS compared to that achieved by the traditional bulk standing wave.

**Figure 3:**
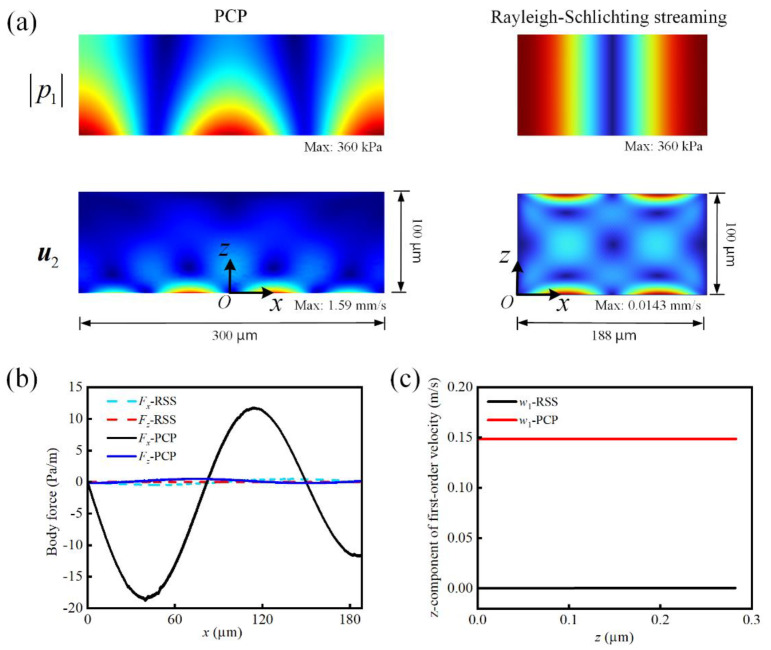
Comparison of the calculated (**a**) acoustic field and AS, (**b**) body force, and (**c**) z-component of the first-order acoustic velocity between the PCP and RSS.

The RSS driven by a bulk standing wave with a driving frequency of 4 MHz was simulated. The boundary condition of the RSS model was adjusted to ensure that the maximum amplitude of the acoustic pressure was the same as that of the PCP resonant field (i.e., 360 kPa), as shown in Figure 2c. The first-order acoustic and second-order AS fields are shown in Figure 3a. The streaming velocity of the PCP-induced AS was two orders of magnitude higher than that of the RSS.

According to the volume force F expressed in Equation (9) for generating AS, we compared the F values resulting from the PCP-induced resonant acoustic field and bulk standing wave. The x- and z-components of F, respectively, denoted as $F_{x}$ and $F_{z}$, along the acoustic boundary layer with thickness $\delta_{vis}=\sqrt{2\mu /\rho_{0}\omega }$ are shown in Figure 3b. Evidently, the $F_{x}$ of the PCP was two orders of magnitude larger than that of the RSS. The other components were at the same level. $F_{x}=-\langle \rho_{0}u_{1}\nabla \cdot u_{1}\rangle -\rho_{0}\langle u_{1}\cdot \nabla u_{1}\rangle =-\rho_{0}\langle u_{1}\nabla \cdot u_{1}\rangle -\rho_{0}\langle u_{1}\partial u_{1}/\partial x\rangle -\rho_{0}\langle w_{1}\partial u_{1}/\partial z\rangle$, where $u_{1}$ and $w_{1}$ are the x- and z-components of the first-order particle velocity, respectively. It was then found that $w_{1}$ of the PCP was three orders of magnitude larger than that of the RSS, which resulted in a larger $F_{x}$ of the PCP, as shown in Figure 3c. This discrepancy originated from the vibration of the PCP in the z-direction. This was missing in the RSS, which was only driven by the standing wave in the x-direction.

### 3.2. Motion of Particles and Separation Efficiency

**Figure 4:**
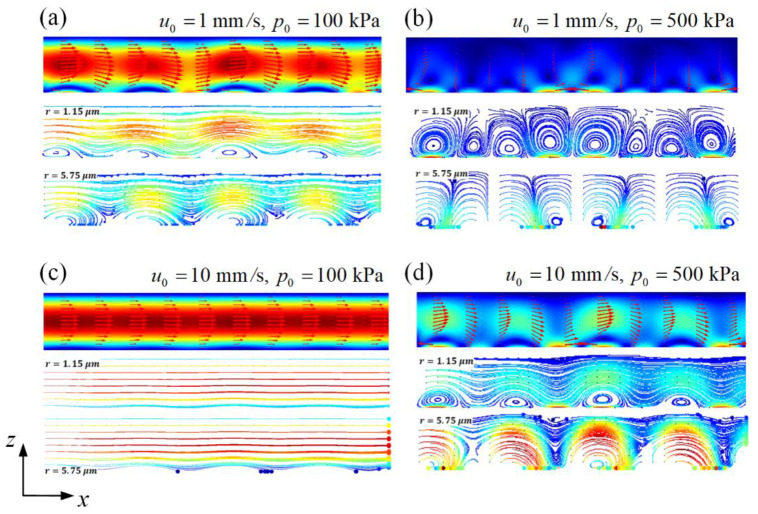
Flow field (top of the figure) and motion trajectories of particles with radii of 1.15 μm (middle of the figure) and 5.75 μm (bottom of the figure) at four different combinations of flow velocities $u_{0}\;$ and incident acoustic pressures $p_{0}$: (**a**) $u_{0}=1\;\mathrm{mm}/s\;\mathrm{and}\;p_{0}=100\;\mathrm{kPa}$ (**b**) $u_{0}=1\;\mathrm{mm}/s\;\mathrm{and}\;p_{0}=500\;\mathrm{kPa}$. (**c**) $u_{0}=10\;\mathrm{mm}/s\;\mathrm{and}\;p_{0}=100\;\mathrm{kPa}$. (**d**) $u_{0}=10\;\mathrm{mm}/s\;\mathrm{and}\;p_{0}=500\;\mathrm{kPa}$.

Studying the effects of the three forces on particle movement is helpful in designing suitable microfluidic devices and operating parameters for particle sieving. First, the motion characteristics of two particles of different sizes A and B under different incident acoustic pressures $p_{0}$ and inlet flow velocities $u_{0}$ are analyzed. The sorted particle size should be determined from the channel size and the forces exerted on the particle in the acoustic field. Particles experience two forces in the acoustic field: the ARF $F_{R}$ and the AS drag force $F_{AS}$. This study relies on the differences in $F_{R}$ on particles of different sizes for particle sieving. The magnitudes of $F_{R}$ and $F_{AS}$ are proportional to the $r^{3}$ and r, respectively. When r decreases, $F_{R}$ decreases more than $F_{AS}$ that gradually dominates the particle motion. According to the calculation, particles smaller than 1 μm have been strongly affected by the AS. In addition, since the height of the channel is 100 μm, the diameter of the particles should not exceed 20 μm to avoid aggregated particles to block the channel. Therefore, in this model, the possible particle size under the driving 4 MHz frequency should be between 1 and 20 μm. Consequently, in this study, the radii of particles A and B were set to 1.15 and 5.75 μm, respectively. It was assumed that the two particles were initially distributed uniformly in the channel, rather than flowing from the inlet. Under the action of $F_{R}$ and $F_{D}$, the particles began to move, as shown in Figure 4. Under the conditions of $u_{0}=1\;\mathrm{mm}/s\;$ and $p_{0}=100\;\mathrm{kPa}$, the AS and inlet flow velocity were similar, and the total flow field distribution after superimposing was non-uniform but remained periodic. The flow was enhanced when the AS and inlet flow had the same flow direction; otherwise, it weakened. The trajectories of the particles showed that most of the A particles flowed out to the channel, which was weakly affected by the ARF. For the B particles, those adjacent to the surface of the PCP were trapped, whereas the upper particles flowed out of the channel. Under these conditions, the two particles underwent significant separation. 

Under the conditions of $u_{0}=1\;\mathrm{mm}/s\;$ and $p_{0}=500\;\mathrm{kPa}$, ARF and AS dominated the particle motion. The A particles followed the streamlines of the AS field, and the B particles were almost trapped on the surface of the PCP. The inlet flow did not have any effect. Although the particles were separated, the A particles accumulated in the cavity and could not be collected. Under the conditions of $u_{0}=10\;\mathrm{mm}/s\;$ and $p_{0}=100\;\mathrm{kPa}$, the acoustic field was weak, and only the B particles close to the PCP were trapped on the PCP surface. Most of these particles flowed out of the channel together with the A particles. Therefore, the particle sieving process exhibited a low separation efficiency. Finally, for the conditions of $u_{0}=10\;\mathrm{mm}/s\;$ and $p_{0}=500\;\mathrm{kPa}$, similarly to the condition of $u_{0}=1\;\mathrm{mm}/s\;$ and $p_{0}=100\;\mathrm{kPa}$, the acoustic and flow fields were relatively balanced, most of the B particles were trapped, while the A particles flowed out of the channel. Overall, to separate the two particles, it is necessary to adjust the ratio of the acoustic and flow fields; a high inlet flow velocity requires a high incident acoustic pressure whereas a low inlet flow velocity requires a low incident acoustic pressure.

**Figure 5:**
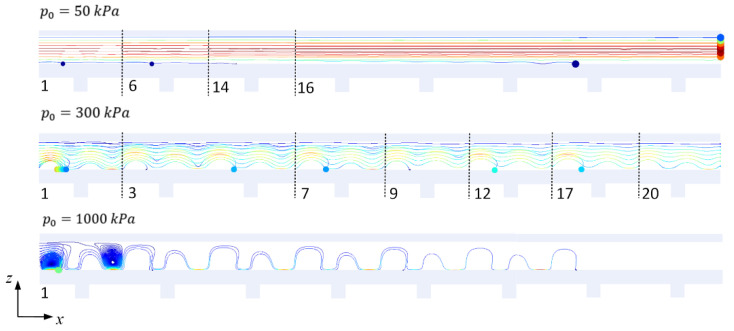
Motion trajectories of particles A and B in the channels under the conditions of $u_{0}=5\;\mathrm{mm}/s$, and $p_{0}=50,\;300$ and 1000 kPa. The length of the channel was 20 times the lattice constant a of the PCP. For a more comprehensible figure, the areas without particles being trapped were omitted. The numbers at the bottom right of the dotted lines represent the actual period number of the lattice to the right of dotted lines.

Secondly, the separation efficiency of the particles flowing from the inlet into the long channel was calculated to simulate real-world conditions. The channel length was set to 20 PCP periods (i.e., 20a=6 mm), and the boundary on both sides was set as a periodic boundary for the acoustic field calculation. In this study, we simulated a situation in which the particles freely flowed into the channel. Under the uniform distribution assumption, we released the uniformly distributed particles at an arbitrary height of the channel at the inlet, as shown in Figure 5. The total number of particles was 2N, and A and B were in equal proportions. We chose a sufficiently long simulation time to ensure that all particles flowed out of the channel and were trapped in the PCP. The number of particles A and B trapped on the PCP surface were $N_{A}$ and $N_{B}$, respectively. The separation efficiency of B was defined as $e_{B}=N_{B}/\left(N_{A}+N_{B}\right)$, which represents the proportion of trapped particles B to all trapped particles. Similarly, the separation efficiency $e_{A}$ of particle A is the proportion of outflow particle A to all outflow particles. According to this definition, the ideal result was $e_{A}=e_{B}=1$. When particles A and B reached complete separation, no mixture occurred. If no particles were trapped, $N_{A}=N_{B}=0$, and $e_{B}=0$. If all particles were trapped, $N_{A}=N_{B}=N$, and $e_{A}=0$. N was set to 10 in this study to balance the simulation time and uniform distribution assumption.

The movement trajectories of the particles under the conditions of $u_{0}=5\;\mathrm{mm}/s$, and $p_{0}=50,\;300\;$ and 1000 kPa are shown in Figure 5. When $p_{0}=50\;\mathrm{kPa}$, most of the particles flowed through the channel, those close to the PCP were trapped, and $e_{A}=0.75$ and $e_{B}=0.56$. When $p_{0}=300\;\mathrm{kPa}$, the B particles close to the PCP were quickly trapped, and the remaining B particles and some A particles close to PCP were trapped over time. Finally, all B particles were trapped, and most A particles flowed out of the channel. Under these conditions, it was calculated that $e_{A}=1.00$ and $e_{B}=0.77$. When $p_{0}=1000\;\mathrm{kPa}$, all particles were almost trapped, small amounts of A particles were involved in the center of the streaming vortex, and $e_{A}=0$. In summary, when $p_{0}=300\;\mathrm{kPa}$, $e_{A}$ and $e_{B}$ achieved higher values. However, because of the height difference between the particles entering the channel, the lower layer of small particles was easily trapped, and it was difficult to achieve complete separation.

**Figure 6:**
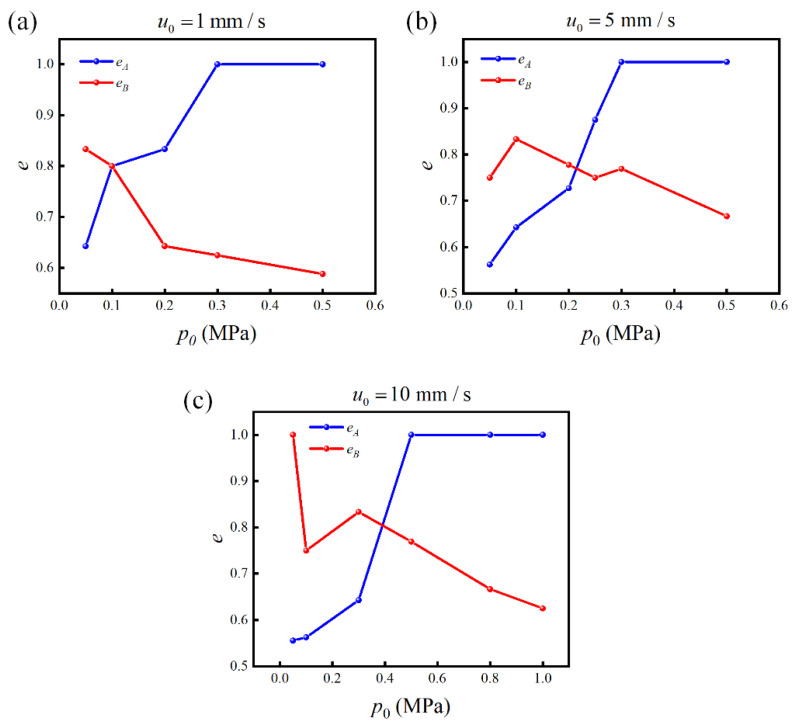
Relationship between the separation efficiencies $e_{A}$ and $e_{B}$ of particles A and B, respectively, with the incident acoustic pressure $p_{0}$ under the flow velocity conditions: (**a**) $u_{0}=1\;\mathrm{mm}/s$. (**b**) $u_{0}=5\;\mathrm{mm}/s$. (**c**) $u_{0}=10\;\mathrm{mm}/s$.

The effects of the different incident acoustic pressures $p_{0}$ on the separation efficiencies $e_{A}$ and $e_{B}$ were further studied under the conditions of $u_{0}=1,\;5\;$ and 10 mm/s. The results are shown in Figure 6. When $u_{0}=1\;\mathrm{mm}/s$, as $p_{0}$ increased, an increasing number of particles A and B were trapped on the PCP surface. Because the number of trapped A particles was larger than that of particles B, the proportion of B was reduced, and $\;e_{B}$ tended to decrease. When all the B particles were trapped, $e_{A}=1$ and $e_{B}=0.63$. Therefore,$\;e_{B}$ was at a low level, and most particles were trapped on the PCP. Overall, it is difficult to determine a suitable $p_{0}$ value under such inlet flow velocity conditions to ensure that $e_{A}\;\mathrm{and}\;e_{B}\;$ reach simultaneously a high level. In the case of $u_{0}=5\;\mathrm{mm}/s\;$ and $p_{0}=50\;\mathrm{kPa}$, only a few B particles were trapped, $e_{A}$ was close to 0.5, and $e_{B}$ was high. As the acoustic pressure increased, an increasing number of B particles were trapped. Simultaneously, a small amount of A particles were trapped, $e_{B}$ decreased briefly, and $e_{A}$ started to rise. When all B particles were trapped, only A particles were present at the outlet. At this time, $e_{A}=1$ and $e_{B}$ reached its second peak, $e_{B}=0.77$. Therefore, for this flow velocity and channel length, $p_{0}=300\;\mathrm{kPa}$ was the best separation parameter, ensuring that both $e_{A}$ and $e_{B}$ could reach high levels. Under the condition of $u_{0}=10\;\mathrm{mm}/s$, $p_{0}=500\;\mathrm{kPa}$ is a more suitable parameter where $e_{A}=1$ and $e_{B}=0.77$. Comparing the results obtained under the three flow rate conditions, it is found that good separation can be achieved for $u_{0}=5\;$ and 10 mm/s. However, under the condition of $u_{0}=1\;\mathrm{mm}/s$, an increase in $e_{A}$ is accompanied by a decrease in $e_{B}$, and it is difficult to reach a higher level. When $e_{A}=1$, the value of $e_{B}$ is smaller than those obtained under the conditions of $u_{0}=5$ and 10 mm/s. Therefore, a low inlet flow velocity is not suitable for particle sieving, possibly because the particles stay in the channel for a longer time under a low flow velocity, and the small particles A are subjected to the radiation force for a longer time. When $p_{0}$ is increased to trap all large B particles, the small A particles are more easily trapped.

### 3.3. Acoustic Separation via PCPs with Different Resonant Frequencies

**Figure 7:**
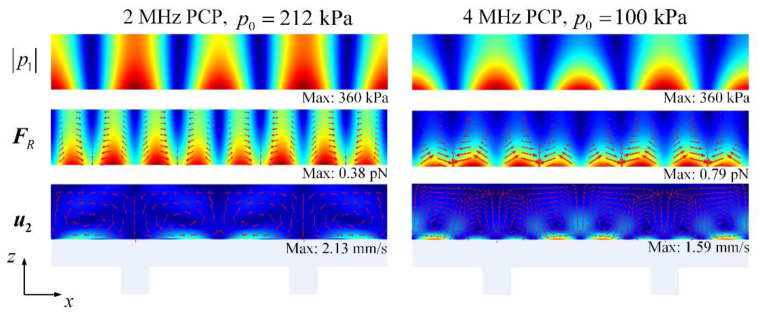
Distribution of the first-order acoustic field $\left|p_{1}\right|$, acoustic radiation force field $F_{R}$, and acoustic streaming field $u_{2}$ for PCPs with resonant frequencies of 2 and 4 MHz. The incident pressures $p_{0}$ are 212 and 100 kPa, respectively.

PCPs with different lattices and thicknesses have different resonant frequencies; therefore, their resonant acoustic fields and induced AS distributions are also different. This section compares the differences between the acoustic fields and AS of PCPs with resonant frequencies of 2 and 4 MHz and discusses their effect on particle sieving. The dimensions of the 2 MHz PCP were h=w=t=100 μm and a=600 μm. Under the same incident acoustic pressure, the acoustic pressure generated from the 2 MHz PCP was lower than that generated at 4 MHz. For the sake of the subsequent comparison, the incident acoustic pressure was adjusted so that the amplitude of the acoustic pressure at the two frequencies was the same, i.e., $p_{0}\left(2\;\mathrm{MHz}\right)=212\;\mathrm{kPa}$ and $p_{0}\left(4\;\mathrm{MHz}\right)=100\;\mathrm{kPa}$. The computed acoustic pressure field, ARF, and AS distributions under these conditions are shown in Figure 7. Because the period of the 2 MHz PCP is longer than that of the 4 MHz PCP, all of the 2 MHz contours were scaled in the x-direction. The distribution of $\left|p_{1}\right|$ reveals that the acoustic field at 2 MHz decays more slowly in the z-direction relative to the 4 MHz acoustic field. Therefore, the field has a wider range of action on the particles. The difference in ARF distribution is similar to that of $\left|p_{1}\right|$, but the ARF generated by the 2 MHz PCP is smaller and is half that of the 4 MHz PCP under an incident acoustic pressure that was approximately two times that of the 4 MHz PCP. The change in the AS distribution was more noticeable. The AS pattern changed between two frequencies, wherein the 2 MHz PCP had two vortices and the 4 MHz PCP had four vortices in one lattice. In addition, the AS caused by the 2 MHz PCP was faster. As shown by these results, under the same acoustic pressure, the low-frequency PCP had a weaker ARF and stronger AS. 

**Figure 8:**
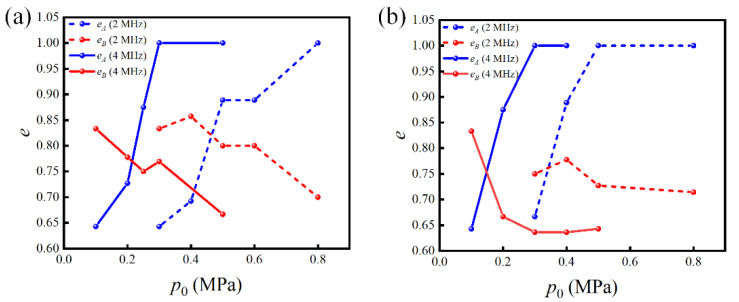
Comparison of the separation efficiency between PCPs with resonant frequencies of 2 and 4 MHz under the conditions of $u_{0}=5\;\mathrm{mm}/s$ and different channel heights of (**a**) 100 and (**b**) 200 μm.

The separation efficiency of the PCPs with resonant frequencies of 2 and 4 MHz was further compared under the conditions of $u_{0}=5\;\mathrm{mm}/s$ and a channel height of 100 μm. The channel length of the 2 MHz PCP was 10 periods, which was consistent with the channel length of the simulation model in the previous section. The results are shown in Figure 8a. It can be seen that 2 MHz PCP exhibits a similar separation efficiency as that achieved at 4 MHz, but at $p_{0}=500\;\mathrm{kPa}$, some particles have already been trapped in the channel under the influence of the AS, as shown in Figure 6. As $p_{0}$ increases, more particles were trapped in the channel, including large particles. Therefore, under this condition, the separation effect at 2 MHz was not as efficient as that at 4 MHz. The results obtained in the previous section indicate that the separation mainly relies on the ARF, and the AS can adversely affect the separation results. Therefore, a low-frequency PCP may not be suitable for sieving particles of small sizes under the conditions investigated in this study. Next, the case in which the channel height was 200 µm was investigated. According to the results, the separation efficiency at 4 MHz was not as good as that at 2 MHz. This is because the acoustic field of the 4 MHz PCP attenuates in the z-direction faster than that of the 2 MHz PCP, making it difficult to trap large particles in the upper part of the channel. Because of the stronger ARF, it is easier to trap small particles near the PCP, resulting in poor separation efficiency.

## 4. Conclusions

Based on the trapping radiation force of PCPs, mixed particles of different sizes can be sieved under continuous flow conditions. Owing to the periodicity of the PCP, these systems have the potential to be used for high-throughput processing and are expected to provide new tools for particle sieving. To find suitable separation parameters and provide theoretical guidance for experiments and for the design of microfluidic devices, we carried out numerical simulations to study the motion of particles under the combined effects of the ARF, AS, and inlet flow conditions. According to the calculations, for different inlet flow velocities, there exists an optimum incident acoustic pressure for achieving the optimum separation efficiency, ensuring the trapping of large particles, and reducing the impact on smaller particles. Moreover, the optimum separation efficiency at a low inlet flow velocity was not as good as that at a high inlet flow velocity. Finally, a high-frequency PCP is more suitable for particle sieving, but the channel height needs to be reduced, which limits the throughput and should be adjusted according to the experimental conditions. In the future, we will fabricate a microfluidic device using a PCP, microfluidic channel with an inlet and outlet, plane transducer, and water cavity to carry out high-throughput particle sieving.

## Data Availability

Not applicable.
